# A Compendium of Materia Medica, Therapeutics and Repertory of the Digestive System

**Published:** 1893-05

**Authors:** 


### BOOK NOTICES AND REVIEWS.

A COMPENDIUM OF MATERIA MEDICA, THERAPEUTICS AND REPERTORY OF THE DIGESTIVE SYSTEM. By Arkell Roger McMichael, M. D. Philadelphia: Bœricke & Tafel, 1892.

Price in cloth, $6.00 net; in half morocco, $7.50 net; expressage extra.

This book is a large and handsome quarto volume. Its plan is unique. The names of the remedies and their symptomatology are arranged in tabular form. The first column on the left-hand page contains the names of the remedies. The second column contains the symptoms of the stomach, each remedy having the symptoms peculiar to itself on the same line with itself. The third column contains appetite and thirst symptoms; the fourth, taste and tongue symptoms; the fifth concomitants; the sixth, mouth and teeth; the seventh nausea and vomiting; the eighth, eructations and flatulence, and the ninth, clinical, and so on. In such an arrangement one reads across the page to get all the symptoms belonging to one remedy. If searching for a certain symptom we have only to run the eye down the column of the anatomical part to which the symptom belongs to find it. Then when we have found it a glance to the left will show to what remedy it belongs, and so much time is saved in hunting the simillimum.

Not content with thus reducing the difficulty of finding the simillimum, the author has added a repertory which is arranged after the manner of a concordance, and the symptom is sought under its most prominent word; nor this alone. *All* the most prominent words of any given symptom are used in placing it in the index, that the difficulty of finding it may be still further reduced. Such painstaking effort cannot be too highly commended in this most industrious author.

From the preface we make the following extract: "The combination of Materia Medica, Therapeutics, and Repertory of a section of the human anatomy will no doubt suggest itself to the busy physician as a decided improvement over the former method of referring to three separate works before deciding on the remedy. Not only has this objection been overcome, but its simple arrangement will appeal to the student as well as to the mature physician; and instead of spending hours in studying our case, a few moments is all that is required (for even the student) to make his choice."

The work being large and weighing five and a half pounds is excluded from the mails and must be sent by express.

